# Acceptance of Psycho-Oncological Counseling Formats in a Cancer Counseling Center during the COVID-19 Pandemic: An Exploratory Care Study

**DOI:** 10.3390/curroncol28050323

**Published:** 2021-09-28

**Authors:** Jacqueline Lohmiller, Norbert Schäffeler, Heike Sütterlin, Stephan Zipfel, Andreas Stengel

**Affiliations:** 1 Section Psychooncology, University Hospital Tübingen, Comprehensive Cancer Center Tübingen Stuttgart, 72076 Tübingen, Germany; jacqueline.lohmiller@med.uni-tuebingen.de (J.L.); norbert.schaeffeler@med.uni-tuebingen.de (N.S.); heike.suetterlin@med.uni-tuebingen.de (H.S.); stephan.zipfel@med.uni-tuebingen.de (S.Z.); 2 Department of Psychosomatic Medicine and Psychotherapy, University Hospital Tübingen, 72076 Tübingen, Germany; 3 Charité Center for Internal Medicine and Dermatology, Department for Psychosomatic Medicine, Charite—Universitätsmedizin Berlin, Corporate Member of Freie Universität Berlin, Humboldt-Universität zu Berlin and Berlin Institute of Health, 10117 Berlin, Germany

**Keywords:** acceptance, office counseling, therapeutic relationship, telephone counseling, video counseling

## Abstract

Background: The COVID-19 pandemic made it necessary to change established structures of medical counseling services and quickly establish digital counseling formats to ensure continuity of care. In this context, we offered telephone and video-telephonic counseling in addition to traditional face-to-face counseling in the office. Methods: Patients (*n* = 100) of the Cancer Counseling Center, Tübingen, were asked to complete a questionnaire to assess the acceptance of the counseling format following each counseling session (office, telephone, and video) in the period between July 2020 and February 2021. The questionnaire included the subject areas of patient characterization, assessment of therapeutic contact, therapeutic relationship, and hurdles and was used in this exploratory care study. Results: The satisfaction and acceptance of the three counseling formats (office, telephone, and video) were rated as “good” to “very good” in the three subgroups (range 1–6, office M = 1.2, telephone M = 1.3, video M = 1.4). Likewise, the “therapeutic relationship” achieved high ratings in terms of establishment of a therapeutic relationship in all three subgroups (office M = 5.7, telephone M = 5.0, video M = 5.0). The type of contact (office and video counseling) achieved a significant main effect on the therapeutic relationship for items such as “I believe that counseling is helping me” (F(2,97) = 4.80, *p* = 0.01) and “I feel that I can rely on the counselor/therapist” (F(2,97) = 3.29, *p* = 0.04). The “hurdles” were rated as minor and tolerable (office M = 1.3, telephone M = 1.3, video M = 1.4). Predictor analyses showed that there was no effect of age and gender on the acceptance of digital counseling formats in the present sample. Discussion and Conclusion: On the basis of this survey, it can be concluded that digital counseling formats were perceived by patients as a promising addition to the classic face-to-face setting. In addition, it can be stated that the digital formats (telephone and video) were not generally preferred to face-to-face counseling, but that the innovative telecounseling was accepted and perceived with great satisfaction and acceptance. Accordingly, the additional use of digital counseling formats could be an opportunity to enrich and expand the existing presence structures also after the COVID-19 pandemic.

## 1. Introduction

The containment measures of the COVID-19 pandemic in 2020 resulted in massive social changes worldwide. This was also the case in the health sector and, in particular, in cancer counseling [1,2,3,4,5,6,7]. The changes caused by the pandemic made it necessary to restructure counseling-specific concepts in order to ensure continuity of support. In the course of conceptual adaptation of established structures in cancer counseling, telephone and videotelephonic counseling formats had to be quickly established [8].

The pandemic confirmed the need to expand and redefine the classic face-to-face meeting and the associated face-to-face office contact. In view of the structural change, the question arose at the outset as to whether relationship building could generally take place in telephonic/video counseling. According to previous studies, the therapeutic relationship is the most stable effective factor of successful perceived support [9,10,11] and represents a significant variable in this context. The principles of action of the therapist–patient relationship are based on different communication channels. Through the use of (video)telephony, limitations such as the “impersonal” perception associated with the elimination of face-to-face interaction between patient and therapist have been described [12]. Another study pointed towards difficulties in mimic and gestural acquisition during telephone and video communication [13]. By eliminating the visual factor in a phone call, it is more difficult to express empathy [14]. Lastly, technical frameworks, through connection problems or distortions, can be disruptive factors in relationship building [15].

In contrast, several studies [16,17,18,19] argue that even through a digital setting, a sustainable therapist–patient relationship can be established, which can be equated to that of a presence-based intervention. In view of the rapid increase in digitalization in society and the need to restructure therapeutic and counseling settings due to the COVID-19 pandemic, the Federal Association of Statutory Health Insurance Physicians opened up legal opportunities to offer digital counseling formats. These allowed traditional locally-based counseling services to become more flexible, time-efficient, and economical through the increased use of telecounseling. The step towards telephonic/video consultations in the Cancer Counseling Center required the establishment of technical foundations that met data protection guidelines.

The aim was to record the acceptance of the offered counseling media, which was surveyed by means of a specially designed questionnaire in a population of patients, in the Cancer Counseling Center. Specifically, the following research questions were addressed: How do patients rate tele-based counseling compared to office face-to-face counseling? Does satisfaction differ according to the counseling setting? Do age and gender have an influence on the acceptance of the different counseling formats?

## 2. Materials and Methods

### 2.1. Procedure

Within the period from July 2020 to February 2021, while the Cancer Counseling Center in Tübingen expanded its counseling services due to COVID-19, an online exploratory survey was conducted on the preceding research questions. All reference therapists were employees of the University Hospital Tübingen. All patients who attended a consultation at the Cancer Center in Tübingen were offered the opportunity to participate in the exploratory care survey. The consultation could be an initial interview contact at the Cancer Counseling Center (26% of respondents), or could occur as part of an ongoing consultation at the Cancer Counseling Center (74% of respondents). Each patient received exactly one questionnaire after a single consultation, or a consultation in the context of ongoing treatment. The participants were asked to complete the questionnaire after the consultation. Only patients who had given their written consent after extensive information and explanation took part in the anonymous data collection. The questionnaire was handed out after each consultation of the three contact formats (office, telephone, and video) in digital form via email. Within the scope of the survey, 121 patients were asked whether they wanted to participate in the survey. These 121 patients were also sent the questionnaire link. In the end, the response rate was 100 patients who returned the completed questionnaire and all of them could be included in the analysis. The study was approved by the local ethics committee (458/2020BO).

### 2.2. Sample

A total of 100 patients (83 women and 17 men) took part in the survey to record judgment and satisfaction regarding the three contact formats offered. In line with the current restrictions of the COVID-19 pandemic, all contact formats were offered that were possible (onsite, telephone, video). Accordingly, patients were asked in the questionnaire which format (digital or onsite) was offered to them. In this context, multiple answers were also possible. The treating therapist with whom counseling took place was randomly assigned to each patient at first contact (psychologist (f)/(m), social pedagogue (f), secretary (f), art therapist (f)). For any followup contacts, the participants had contact with the therapist they had contacted at the first contact. The following analyses are based on the descriptive and psychometric data collected through exploratory data collection using a specially designed questionnaire.

### 2.3. Survey

The questionnaire was constructed in our team and internally validated. The questionnaire consisted of four subject areas, namely: “patient characterization” (12 items, multiple choice), “assessment of therapeutic contact” (14 items, 6 point Likert scale, different categories such as “personal” to “impersonal”), “therapeutic relationship” (11 items, 6 point Likert scale, categories “very inapplicable” to “very applicable”) and “hurdles” (5 items, 6 point Likert scale, categories “I do not agree at all” to “I fully agree”).

In addition, we asked 5 free-text items about reasons for choosing the type of contact and about feedback, (e.g., “Is there anything you particularly liked or disliked about the counseling/counseling center?”).

The construction of the “assessment of therapeutic contact” subject area was based on the predictors “global judgment”, “experience of the contact”, “satisfaction of the counseling session”, and “satisfaction with the general conditions”. The “therapeutic relationship” inquired about the assessment of the relationship with the counselor/therapist. Finally, the “hurdles” subject area recorded the subjective perception with regard to the “technique” or the “framework conditions” and, if applicable, the instructions for safety (mouth–nose protection, disinfection, distance, etc.). We used an ad hoc questionnaire, which was designed specifically for the current study.

### 2.4. Statistical Analyses

The examination of the questions was performed using IBM SPSS Statistics for Windows, version 27 (IBM Corp., Armonk, NY, USA). Sociodemographic characteristics were analyzed descriptively. Hierarchical regression analyses were performed to examine the relationships between age or gender and three dependent variables (“assessment of therapeutic contact” “therapeutic relationship”, and “hurdles”). No outliers were found in the analysis of the standardized residuals. In addition, checks for violations of the assumptions of collinearity, independent error, normal distribution, homoscedasticity, and linearity were also performed. In this context, all assumptions were met.

## 3. Results

The study population consisted of 100 patients (83 women, 17 men). The mean age was 47.5 years (range = 18–80 years). Of the 100 participants, 44% of the counseling contacts occurred via the face-to-face format, 24% via the telephone format, and 32% via the video format (Table 1). In addition, in 57% the contact took place with a female psychologist, in 19% with a male psychologist. In total, 15% stated that the consultation took place with a social pedagogue (female), 5% with the secretariat (female; this contact was not surveyed as a therapeutic contact, but can be seen as a remembered contact in the context of the outpatient presentation), and 4% with an art therapist (female, Table 1). Within the present sample, 26% reported the contact as first contact, while 74% indicated that the contact took place as contact within an ongoing consultation (Table 1).

**Figure 1:**
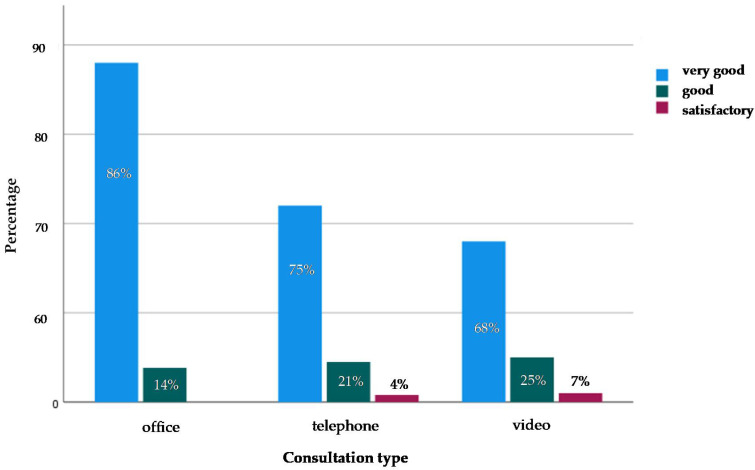
Satisfaction with the type of contact. Percentages (%) as cumulative values per category.

The assessment of satisfaction comparing the three counseling formats showed on average a high level of satisfaction (Figure 1). The contact during “face-to-face” counseling was rated as very good in 86% of the cases and good in 14% of the cases. Telephone contact scored very good in 75% of cases, good in 21% of cases, and satisfactory in 4% of cases. Video contact was rated very good in 68% of cases, good in 25% of cases, and satisfactory in 7% of cases (Figure 1). By first glance, face-to-face counseling was rated very well and seemed slightly preferred over telephone or video counseling. Therefore, in the next step a detailed assessment of the subject areas “assessment of therapeutic contact”, “therapeutic relationship”, and “hurdles” was performed.

Within the subject area “assessment of therapeutic contact”, the item “global judgment” had a mean of 1.3 (SD = 0.5) in the overall group. In subgroup office, the mean value ± SD of this item was 1.1 ± 0.3, in the telephone subgroup, 1.3 ± 0.6 and in the video subgroup, 1.4 ± 0.6 (F(2,97) = 2.28, *p* = 0.11, Table 2). All other items assessed were in the range of M = 1.1 to 1.6, both for the overall group and within the three subgroups, i.e., all were rated between very good and good. No significant differences between subgroups were observed (Table 2).

“Therapeutic relationship” was rated “true” to “very true” with mean values of items ranging between 3.9 and 5.4 in the overall group (Table 2). Subgroup office scored “true” to “very true” for items of the “therapeutic relationship” with mean scores ranging from 4.0 to 5.8. A similar picture was observed for the telephone (3.9 to 5.3) and video (3.8 to 5.3) subgroups (Table 2).

The type of contact (office and video counseling) had a significant main effect on the therapeutic relationship for the items: “I believe that my counselor/therapist is helping me” (F(2,97) = 4.17, *p* = 0.02), “I believe that counseling is helping me” (F(2,97) = 4.80, *p* = 0.01), “I have gained some new insights” (F(2,97) = 4.47, *p* = 0.02), and “I feel that I can rely on the counselor/therapist” (F(2,97) = 3.29, *p* = 0.04; Table 2). The following items showed a main effect with regard to the contact types office and telephone counseling: “I feel that the counselor/therapist understands me” (F(2,97) = 5.02, *p* = 0.01), “I feel that the counselor/therapist wants me to achieve my goals” (F(2,97) = 3.31, *p* = 0.04), “I feel that I, as well as the counselor/therapist, are seriously pulling together” (F(2,97) = 5.02, *p* = 0.01), “I feel that I and the counselor/therapist see and assess my problems similarly” (F(2,97) = 3.31, *p* = 0.04), and “I feel that I can now understand myself and deal with myself independently” (F(2,97) = 5.02, *p* = 0.01; Table 2).

The “hurdles” were rated as “low” (1.3 ± 0.6) on average in the overall group assessing the item “The necessary technology/framework conditions overwhelmed me”. The same picture emerged in all three subgroups: office (1.3 ± 0.5), telephone (1.3 ± 0.6), and video (1.3 ± 0.6). Not significant differences between subgroups were observed for this or other items of the “hurdles” subject area (*p* > 0.05, Table 2).

Within the three subordinated subject areas, a regression analysis did not show a significant effect of age or gender on “assessment of therapeutic contact” (age: F(5, 83) = 0.840, *p* = 0.525; gender: (F(1,87) = 0.376, *p* = 0. 541), “therapeutic relationship” (age: (F(5,94) = 0.944, *p* = 0.457, gender: (F(1,98) = 0.018, *p* = 0.895) and “hurdles” (age: (F(1,98) = 0.018, *p* = 0.895, gender: (F(1,87) = 0.145, *p* = 0.704).

## 4. Discussion

The COVID-19 pandemic in 2020 made it necessary to break up established structures of medical counseling services and establish digital counseling formats in order to ensure continuity of care. In this context, the Cancer Counseling Center Tübingen reacted quickly to the pandemic-related changes and offered telephone and videotelephonic counseling in addition to traditional “face-to-face” office counseling. Therefore, the question arose whether digital counseling services (telephony or videotelephony) were equally accepted compared to the traditional face-to-face setting.

For this explorative study, a specially designed questionnaire was used, which was oriented towards the subject areas “patient characterization”, “assessment of therapeutic contact”, “therapeutic relationship”, and “hurdles”.

Congruent with results of previous research [16], all types of contact were assessed with very high or high acceptance. This evaluation was underpinned qualitatively by the written free-text modules, which stated that telephone and videotelephonic counseling could open up more room for “flexible time and conversation arrangements”.

With regard to the “therapeutic relationship”, a very homogeneous picture emerged within the subgroups (office, telephony, and video). The relationship was rated significantly as very understanding and appreciative both in the face-to-face setting and within the digital formats. However, some items were rated slightly (but still significantly better) with respect to the digital formats, such as “I believe that my counselor/therapist is helping me”, “I believe that counseling is helping me”, “I have gained some new insights”, and “I feel that I can rely on the counselor/therapist” in office and video counseling. As well as the items “I feel that the counselor/therapist understands me”, “I feel that the counselor/therapist wants me to achieve my goals”, “I feel that I, as well as the counselor/therapist, are seriously pulling together”, “I feel that I and the counselor/therapist see and assess my problems similarly”, and “I feel that I can now understand myself and deal with myself independently” in office and telephone counseling. Despite this, it’s useful to note that in-office contacts scored slightly better in terms of relationship building (Table 2).

This could point towards successful and stabilizing relationship building both in the context of online formats and in the context of office settings. Nonetheless, it can be stated that it seems possible to build a trusting relationship even without the physical presence. These results confirm the findings of previous research [17,18,19] and refute the assumption that therapeutic relationship-building is solely based on present and direct contact between therapist and patient [12].

In addition, according to the present study, videocounseling has the potential, when conducted within the “familiar and domestic” environment, to create a “safe environment”, which is perceived as “trusting” and “relaxing” in our study. Moreover, the counseling session in front of the home computer or at home on the phone, likely reduced the fear of contracting COVID-19 within an office setting. In the subgroup comparison, the “hurdles” in the video and telephone groups were also rated only slightly (and not significantly) higher than in the face-to-face consultation. Exclusively technical complications were rated as partially “disturbing” or “hindering” in the video setting but did not differ significantly. Interestingly, age or gender did not have a significant impact on the evaluation indicative of the acceptance and broad agreement regarding psycho-oncology telecounseling across gender and a broad age range (18 to 80 years).

As a limitation, the present pilot study was conducted in a cross-sectional manner in a selected and rather small population of patients. Therefore, generalized statements should be made with caution. However, the findings obtained are encouraging and should be repeated and validated in other patients’ groups, larger populations, and also in a longitudinal manner.

Due to the fact that the survey was conducted during the COVID-19 pandemic, it should be noted that distance as well as hygiene rules had to be observed and mouth–nose protection had to be worn. These circumstances may have had an impact on the patients’ assessments and should be assessed and evaluated in further studies in the future.

The following future directions could be subsumed under the limitations already mentioned. Future studies should pay attention to a sufficiently large sample in order to be able to statistically verify the acceptance of the offered counseling media even better. Furthermore, the perception of the COVID-19 restrictions by the mouth–nose protection, or the distance rules should be surveyed and compared with “restriction-free” sessions. Another point would be the assessment of the treating therapists, which should be included in the survey in order to be able to capture the change in perspective of the therapeutic relationship. Finally, it should be noted that the professional background of the treating therapist could have an influence on the final satisfaction of the patient and should therefore be addressed in further studies.

Considering the fact that there is already some evidence for the acceptance and effectiveness of video-based and telephone counseling formats [8,15,19], this exploratory care study was able to illustrate that especially in cancer counseling, video-telephonic counseling can assume an essential and effective support function.

## 5. Conclusions

Taken together, it can be concluded that digital counseling formats were perceived as a promising addition to the traditional face-to-face office setting and perceived with great satisfaction and acceptance. In addition, digital formats (telephone and video) were neither perceived as inferior nor generally superior to face-to-face office counseling. An important aspect of the study was to look at how digital counseling formats were evaluated and adopted during the pandemic. Future studies should therefore take a closer look at telecounseling across several counseling sessions and against the background of different diagnoses and counseling services and also after the pandemic. It is very likely that telecounseling will remain in place and provide an important addition also in the post COVID-19 era in cancer counseling.

## Figures and Tables

**Table 1:** Descriptive data.

Characteristic		Mean	%
Age		47.5 (years)	
Gender			
	Male		17
	Female		83
Contact type			
	Telephone consultation		24
	Video consultation		32
	Office consultation		44
Treating therapist			
	Psychologist (f)		57
	Psychologist (m)		19
	Social pedagogue (f)		15
	Secretariat (f)		5
	Art therapist (f)		4
Contact occasion			
	First contact		26
	Contact within ongoing consultation		74

Description of the study population (*n* = 100). Abbreviations: f = female, m = male.

**Table 2:** Assessment of therapeutic contact, therapeutic relationship, and hurdles in different counseling formats.

	All Contacts	Office	Telephone	Video	df	F	*p*
**Assessment**							
global judgment conversation contact	1.3 ± 0.5	1.1 ± 0.3	1.3 ± 0.6	1.4 ± 0.6	2	2.28	0.11
Personal—impersonal	1.2 ± 0.5	1.1 ± 0.3	1.3 ± 0.6	1.3 ± 0.5	2	1.18	0.31
pleasant—unpleasant	1.2 ± 0.6	1.2 ± 0.4	1.3 ± 0.8	1.3 ± 0.6	2	0.32	0.73
friendly—unfriendly	1.1 ± 0.3	1.0 ± 0.2	1.1 ± 0.4	1.1 ± 0.3	2	0.56	0.57
suitable—unsuitable	1.2 ± 0.5	1.2 ± 0.4	1.4 ± 0.7	1.2 ± 0.5	2	2.19	0.12
helpful—not helpful	1.4 ± 0.8	1.3 ± 0.5	1.5 ± 0.7	1.5 ± 1.1	2	1.28	0.28
understanding—not understanding	1.2 ± 0.4	1.1 ± 0.3	1.2 ± 0.7	1.2 ± 0.4	2	0.20	0.82
supportive—not supportive	1.3 ± 0.6	1.2 ± 0.5	1.2 ± 0.7	1.4 ± 0.8	2	0.68	0.51
empathic—not empathic	1.3 ± 0.5	1.2 ± 0.5	1.3 ± 0.7	1.3 ± 0.4	2	0.11	0.89
I felt comfortable—I felt uncomfortable.	1.3 ± 0.6	1.2 ± 0.4	1.4 ± 0.9	1.3 ± 0.6	2	1.18	0.31
I could open up well—I could not open up well.	1.3 ± 0.6	1.2 ± 0.5	1.4 ± 0.7	1.4 ± 0.8	2	1.08	0.35
I fully trust my counselor/therapist—I cannot trust my counselor/therapist.	1.4 ± 0.7	1.2 ± 0.6	1.6 ± 1.1	1.3 ± 0.7	2	2.28	0.11
My concern and my problem situation were comprehensively inquired about and understood—my concern and my problem situation were insufficiently inquired about and understood.	1.3 ± 0.7	1.2 ± 0.6	1.3 ± 0.8	1.4 ± 0.7	2	0.84	0.44
satisfaction of counseling interview	1.3 ± 0.6	1.2 ± 0.4	1.3 ± 0.7	1.4 ± 0.7	2	1.31	0.28
**Therapeutic Relationship**							
I believe that my counselor/therapist is helping me.	5.3 ± 1.3	5.7 ± 0.6	5.0 ± 1.7	5.0 ± 1.5	2	4.17	0.02
I believe that counseling is helping me.	5.3 ± 1.3	5.7 ± 0.5	4.9 ±1.6	4.9 ± 1.5	2	4.80	0.01
I have gained some new insights.	5.1 ± 1.3	5.5 ± 0.7	4.9 ± 1.4	4.7 ± 1.6	2	4.47	0.02
I have recently started to feel better.	4.5 ± 1.2	4.7 ± 1.1	4.6 ± 1.2	4.2 ± 1.3	2	1.74	0.18
I can already foresee that I may be able to overcome the problems I came to counseling for.	4.3 ± 1.4	4.3 ± 1.3	4.2 ± 1.4	4.2 ± 1.3	2	0.14	0.87
I feel that I can rely on the counselor/therapist.	5.3 ± 1.2	5.7 ± 0.6	5.1 ± 1.7	5.1 ± 1.3	2	3.29	0.04
I feel that the counselor/therapist understands me.	5.4 ± 1.2	5.8 ± 0.6	4.8 ± 1.7	5.3 ± 1.4	2	5.02	0.01
I feel that the counselor/therapist wants me to achieve my goals.	5.4 ± 1.1	5.7 ± 0.6	5.0 ± 1.6	5.3 ± 1.2	2	3.31	0.04
I feel that I, as well as the counselor/therapist, are seriously pulling together.	5.3 ± 1.1	5.7 ± 0.6	4.8 ± 1.6	5.2 ± 1.2	2	5.02	0.01
I feel that I and the counselor/therapist see and assess my problems similarly.	5.1 ± 1.2	5.5 ± 0.7	4.5 ± 1.5	5.1 ± 1.3	2	3.31	0.04
I feel that I can now understand myself and deal with myself independently.	3.9 ± 1.3	4.0 ± 1.3	3.9 ± 1.4	3.8 ± 1.3	2	5.02	0.01
**Hurdles**							
satisfaction framework conditions	1.3 ± 0.6	1.3 ± 0.5	1.3 ± 0.6	1.3 ± 0.6	2	0.06	0.94
The necessary technology/framework conditions overwhelmed me.	1.6 ± 1.3	1.5 ± 1.4	1.3 ± 0.7	1.8 ± 1.6	2	0.77	0.47
The necessary technology/framework conditions were very distracting to me.	1.5 ± 1	1.6 ± 1.2	1.4 ± 0.8	1.5 ± 0.8	2	0.31	0.73
I was able to fully concentrate on the content of the conversation.	4.0 ± 2.2	4.1 ± 2.2	3.2 ± 2.2	4.3 ± 2	2	1.97	0.15
I was worried about catching a cold.	1.3 ± 1	1.4 ± 1	1.6 ± 1.3	1.1 ± 0.5	2	1.83	0.17
I was worried about doing something wrong.	1.5 ± 1.2	1.5 ± 1.3	1.5 ± 1.1	1.5 ± 1	2	0.06	0.94

## Data Availability

The data presented in this study are available on request from the corresponding author.
